# New Year's Eve in otorhinolaryngology: a 16-year retrospective evaluation

**DOI:** 10.1007/s00405-023-07966-2

**Published:** 2023-04-17

**Authors:** Julia Werz, Jens Greve, Thomas K. Hoffmann, Janina Hahn

**Affiliations:** 1 Department of Otorhinolaryngology, Head and Neck Surgery, University Hospital Ulm, Frauensteige 12, 89075 Ulm, Germany; 2 Division of Phoniatrics and Paediatric Audiology, Waldstr. 1, Erlangen, Germany

**Keywords:** New Year’s Eve, Otorhinolaryngology, Fireworks, COVID-19

## Abstract

**Purpose:**

Pyrotechnics are a long-standing tradition at the turn of the year. There are little data available on New Year's Eve-associated ORL injuries. Due to restrictions during the Corona pandemic, the handling of fireworks and meetings on New Year's Eve 2020–2022 had been significantly changed. Our aim was to analyze first data about New Year's Eve-associated ORL injuries.

**Methods:**

A retrospective analysis of 16 turns of the year (2006–2022) at a University ORL department was performed. The 2 recent years were influenced by the changes and restrictions of the COVID-19 pandemic.

**Results:**

Of 343 emergency presentations, 69 presented with New Year's Eve-associated reasons (20%). 72% were male, 15.9% were underage. 74% presented for fireworks-related injuries, 19% due to violent altercations. Noise trauma was present in 71%. The average number of New Year's Eve-associated emergency patients per year and the average total number of patients were reduced by more than half under COVID-19 pandemic conditions.

**Conclusions:**

New Year's Eve-associated ORL injuries range from inner ear trauma to midface fractures. Long-term damage may include hearing loss and tinnitus. These results shall support the responsible use of fireworks even after the end of the special regulations of the COVID-19 pandemic.

## Introduction

For many decades, fireworks of category F2 and other pyrotechnic objects like firecrackers have traditionally been set off by private individuals on New Year's Eve in Germany. The category F2 includes small fireworks such as bangers and firecrackers that are not intended to be too dangerous, have a comparatively low noise level and are intended for the use in confined outdoor areas. Fireworks articles of category F2 may have a maximum noise level of 120 dB at a distance of 8 m [1]. Sound pressure peaks above 150 dB can lead to a blast or explosion trauma, depending on the duration of exposure. If the sound pressure peaks are present for at least 2 ms, explosion trauma can occur. Consequences may include rupture of the tympanic membrane, possibly with dislocation and/or fracture of the auditory ossicles and hemorrhage into the tympanic mucosa. In case of blast and explosion trauma, also a hearing loss of varying severity results.

For the turn of the years 2020/2021, as well as for 2021/2022, a nationwide ban on the release of fireworks of category F2 was declared in Germany. The reason for this was that, due to the COVID-19 pandemic, hospitals should not be burdened with further emergencies caused by fireworks-injured patients. Some federal states and cities established further individual rules for handling pyrotechnics: in the states of Baden-Württemberg and Bavaria, for example, fireworks and firecrackers were only permitted on private property due to the curfew at night. An analysis by a health insurance company showed that during the COVID-19 pandemic, the number of hospital admissions due to New Year's Eve accidents nationwide dropped from about 6.200 cases in 2019 to about 3.800 cases the following year [2]. In addition to the ban on fireworks, there was an influence at the turns of the year during the COVID-19 pandemic due to various other measures as part of the lockdown. These included contact restrictions at private gatherings, the closure of nightclubs, the ban on fireworks in central locations and the curfew.

Pyrotechnics can cause serious injuries, especially in the context of non-professional private use. Mainly reports and studies on injuries of the eyes and hands are published [3–14]. A study from the Netherlands reports that burns of the upper extremities and eye injuries are the most common injuries caused by pyrotechnics on New Year's Eve, especially among adults [15]. Studies of ear, nose and throat (ENT) injuries from pyrotechnics on New Year's Eve are scarce in the literature to date.

The aim of our monocentric retrospective study was to evaluate all emergency patients presenting to the ENT-Department of a University hospital in Southern Germany on New Year's Eve. The intention was not only to obtain an analysis of ENT injuries caused by fireworks on New Year's Eve, but also to determine the partly controversially discussed influence of the above-mentioned restrictions during the COVID-19 pandemic on emergency presentations in the ENT-Department.

## Materials and methods

A retrospective analysis of all patients who presented as emergencies at an ENT-University hospital department in Southern Germany was performed over a period of 16 turns of the year (2006–2022). All patients (no restriction regarding age and gender) who arrived between 4:00 pm on December 31st and 3:59 pm on January 1st were included. This time period was chosen by the authors to cover, as far as possible, the actual effects of New Year's Eve. Therefore, a 24-h period was chosen, starting in the afternoon. Outpatients were evaluated as well as patients who were admitted as inpatients after the emergency presentation. The presentations were divided into New Year's Eve-associated and New Year's Eve-unrelated. New Year's Eve-associated presentations were further categorized into the following three causes: Fireworks, violent altercation, and other New Year's Eve-associated trauma. The basis for the categorization was the medical documentation of the incident. Physical violence that occurred for example during New Year's Eve celebrations or while drinking alcohol was categorized as New Year's Eve associated. The clinical courses of all patients were further analyzed with respect to the need for imaging and/or surgical intervention. Other patient-specific characteristics such as age and gender were considered. Patients who had reached the age of 18 years were assigned as of legal age.

In addition, a comparison was made between the 14 turns of the year with "usual" handling of pyrotechnics, as well as the two turns of the year under the ban on the transfer of fireworks of category F2 and the upper mentioned rules during the pandemic. The basis for the retrospective evaluation were the medical documentation of the electronic patient records of the department. The analysis of the data was mainly performed descriptively.

## Results

### Patient characteristics and numbers of emergency presentations

Of a total of 343 emergency presentations between 4:00 pm on December 31st and 3:59 pm on January 1st from 2006 to 2022, 69 of the patients presented for New Year's Eve-associated reasons (20%). 50 of the 69 patients were male (72%) and 19 were female (28%). The average age of the patients was 31.6 years (range 3–76 years). 11 of the 69 patients (15.9%; 9 male, 2 female) were underage.

51 of the 69 patients (74%) presented because of fireworks injuries and 13 patients presented because of violent altercations (19%). 5 patients (7%) presented due to other New Year's Eve-associated causes; these are listed in Table 1.

**Table 1:** Other New Year's Eve-associated causes for emergency presentation (*n* = 5) from 2006 to 2022 between 4:00 pm, December 31st and 3.59 pm, January 1st

Other New Year's Eve-associated causes for emergency presentation (*n* = 5)
1. Nasal bone fracture due to a hit while dancing
2. Open nasal bone fracture due to a fall caused by alcohol intake
3. Fall on a New Year's Eve celebration leading to a strongly bent fixation of the patient’s epithesis
4. Tympanic membrane perforation due to insertion of a bending light into the ear canal
5. Acute acoustic trauma by an alarm pistol

In terms of absolute numbers, the 2009–2010 turn of the year was the one with the most New Year's Eve-associated presentations (*n* = 10), and the 2020–2021 turn of the year, influenced by the COVID-19 pandemic, was the one with the fewest New Year's Eve-associated emergency presentations (*n* = 0). At the turns of 2014–2015 and 2018–2019, the total number of emergency presentations was highest (n = 29), and from 2021 to 2022 (again under the influence of the COVID-19 pandemic) was the lowest total number of emergency patients (*n* = 4). Table 2 lists all absolute and relative numbers of emergency patients.

**Table 2:** Overview of the absolute and relative number of New Year’s Eve-emergency presentations from 2006 to 2022

Year	Number of patients New Year’s Eve-associated/Total number of emergencies	Proportion (%) of New Year’s Eve-associated patients	Number (n) of female New Year’s Eve-associated patients
2006–2007	2/16	12.5%	0
2007–2008	3/20	15%	1
2008–2009	4/14	28.6%	1
2009–2010	11/28	39.3%	6
2010–2011	2/12	16.7%	0
2011–2012	5/25	20%	0
2012–2013	7/28	25%	3
2013–2014	4/24	16.7%	0
2014–2015	1/29	3.4%	0
2015–2016	10/27	37%	4
2016–2017	4/23	17.4%	2
2017–2018	3/25	12%	0
2018–2019	7/29	24.1%	0
2019–2020	5/21	23.8%	2
2020–2021	0/18	0%	0
2021–2022	1/4	25%	0

### Diagnosis and therapy

**Fig. 1:**
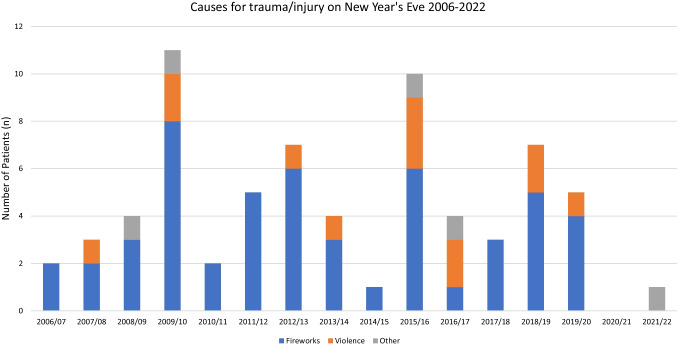
Causes for New Year’s Eve-associated emergency presentations from 2006 to 2022 (In total *n* = 69)

A further analysis was performed with regard to the injuries sustained (Fig. 1). Noise trauma was present in 49 of the 69 New Year's Eve-associated emergency patients (71%). A burn was present in one patient (1.5%). Tympanic membrane perforation was diagnosed in 7 of the 69 patients (10.1%). All diagnoses are listed in Table 3, where multiple diagnoses could be made in one patient.

**Table 3:** All diagnoses of patients with New Year’s Eve-associated emergency presentation

Diagnosis/Symptom	Number of patients	Proportion (%) of *n* = 69
Tinnitus	38	55%
Hearing loss	35	50.7%
Soft tissue injury	8	11.6%
Nasal bone fracture	7	10.1%
Perforation of the ear drum	7	10.1%
Fracture of the midface	4	5.8%
Bruised nose	4	5.8%
Epistaxis (after trauma)	2	2.9%
Hyperacusis	2	2.9%
Burn	1	1.4%
Calvarian skull fracture	1	1.4%
Intracerebral hemorrhage	1	1.4%
Bent fixation of the patient’s epithesis	1	1.4%

Total number of patients *n* = 69, multiple diagnoses in one patient possible

**Fig. 2:**
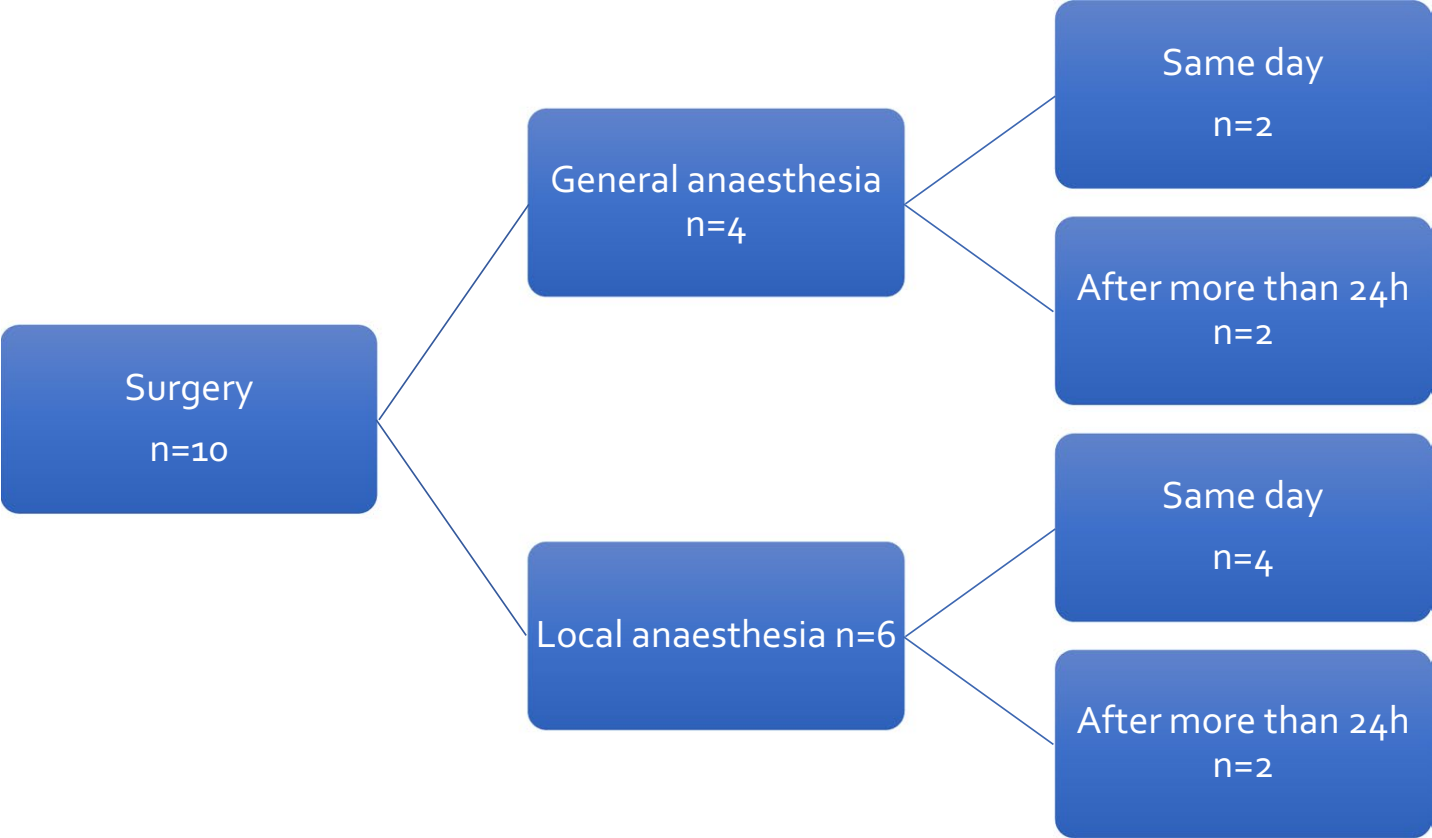
Clinical course of patients (*n* = 10), with New Year’s Eve-associated Trauma who needed surgical intervention. 2006–2022; *h* hours

Surgical care had to be provided in 10 of the 69 New Year's Eve-associated emergency patients (14.5%). Surgical care was defined as any surgical intervention, both under local and general anesthesia. Interventions under local anesthesia included for example sutures and tympanic membrane splinting. An overview of the interventions is shown in Fig. 2.

Inpatient admission was required in 4 of the 69 New Year's Eve-associated emergency patients (5.8%).

Imaging was performed for New Year's Eve-associated injury in 7 of the 69 patients (10.1%), in all cases, CT scan was the method of choice.

### Influence of the COVID-19 pandemic

A total of 68 patients (21.2%) presented for New Year's Eve-associated injuries at the 14 turns of the year 2006/2007 to 2019/2020 which were not yet impacted by regulations during the COVID-19 pandemic. The total number of emergency presentations during this period was 321. On average, this equates to 5 patients due to New Year's Eve-associated injuries annually for an average total of 23 emergency presentations between 4:00 pm on December 31st and 3:59 pm on January 1st.

Both turns of the years 2020/2021 and 2021/2022 were during the COVID-19 pandemic, and thus affected by the measures mentioned in the introduction. There were no New Year's Eve-associated emergency presentations at the turn of 2020/2021 for a total of 18 patients presenting as emergencies during the period studied. The turn of the year 2021/2022 was characterized by the lowest of the total number of patients (*n* = 4), with only one New Year's Eve-associated emergency presentation, due to noise trauma caused by a scare gun. Overall, there was one New Year's Eve-associated emergency presentation of 22 patients at the turns of the year during the COVID-19 pandemic during the period studied (4.5%) which averages to 0.5 New Year's Eve-associated emergency presentations per turn of the year for an annual average of 11 total emergency patients. Table 4 summarizes the comparison.

**Table 4:** Comparison of the turns of the year before the COVID-19 restrictions (2006/2007–2019/2020) and during COVID-19 (2020/2021–2021/2022). Study period: 4:00 pm on 12/31 to 3:59 pm on 01/01

	New Year’s Eve before COVID-19 (2006/2007–2019/2020)	New Year’s Eve during COVID-19 (2020/2021–2021/2022)
Proportion (%) of New Year’s Eve-associated patients	21.2%	4.5%
Average number 0f New Year’s Eve-associated number of patients per year	5	0.5
Average number of emergency patients during the study period	23	11

## Discussion

Previous data on studies of New Year's Eve-associated injuries in ENT are scarce. There are a few publications on pyrotechnic injuries from general emergency departments and especially with a focus on Ophthalmology and Hand Surgery. Nevertheless, the present data show that otolaryngology is clearly affected by the effects of New Year's Eve behavior, particularly through acoustic trauma and midface fractures.

In our study of a Southern German University ENT emergency Department, 50 of the 69 patients with New Year's Eve-associated injuries leading to presentation to the emergency department were male. This percentage of 72% is consistent with data from New Year's Eve analyses from other disciplines, which also report a dominant proportion of male patients (Department of Ophthalmology: 71.2%, systematic review from Ophthalmology: 75%—range: 66–95% [9, 14]). The percentage of male patients in a study of hand injuries from fireworks was even higher at 96.6% [10]. Similarly, the percentage of male patients in a retrospective evaluation of severe fireworks injuries that resulted in hospital admission or surgery was over 90% [16]. The number of underaged patients in the present study at 15.9% was lower compared to studies from ophthalmology (Lenglinger 2021: 34.9% [9]).

The most common cause of ENT injury was inner ear trauma from fireworks. The patterns of injury from pyrotechnics were wide-ranging, from tympanic membrane perforations to noise trauma. Burns were rare in the present study, with only one affected patient. In comparison, burns were the most common injury from fireworks at 48% (*n* = 38) in a prospective multicenter study from the Netherlands which recruited from a specialized burn center in addition to general emergency departments [15]. It should also be noted, in comparison to other studies, that the present evaluation did not include only fireworks-induced injuries. The rate of patients requiring surgical treatment (14.5%) was nevertheless comparable to other studies (van Yperen et al.: 20% [15]). One limitation of our study is that sound audiometry is not regularly recorded in emergency patients. Thus, only the presence of an acute noise trauma with mostly subjective hearing loss and in most of the cases the results of tuning fork tests (of Weber and Rinne) was documented. In most of the cases no statement could be made about the severity of the symptoms and the further clinical course. This is partly due to the fact that most patients returned to their local ENT specialist for further follow-up, which was not possible on December 31st and January 1st due to opening hours. Another point of discussion can be the analysis of the patients after alcohol consumption or physical confrontation in this context. It is obvious that this is not a circumstance that can be attributed to New Year’s Eve alone, but also occurs on “normal” weekends. Nevertheless, an increased alcohol consumption is certainly present on this night, so that an association with the festivities cannot be dismissed out of hand.

One important topic to discuss in a monocentric analysis is the influence and availability of nearby emergency departments and ENT specialists. During the inclusion criteria time (between 4:00 pm on December 31st and 3:59 pm on January 1st), ENT emergency departments and/or ENT specialists are rarely available in a surrounding area of about 100 km radius except one other ENT department in the same town. This might explain the relatively small number of violence-induced injuries compared to symptoms or injuries of the inner ear, as the first-mentioned, like soft tissue injury, is also treated in some general emergency departments without ENT specialists. The treatment spectrum and availability of other involved disciplines such as Pediatrics or Oral and Maxillofacial Surgery also have a relevant influence and the local structures must be considered in this monocentric evaluation: The Pediatric Department is located next door, children with injuries of the head and neck are usually send to and treated in the ENT department. The Department of Oral and Maxillofacial Surgery with its emergency unit is located in the same town. Nasal bone fractures and fractures of the midface without involvement of the mandible or occlusion are frequently treated in the ENT department. In addition to the last-mentioned discussion points, it must be considered that an unknown number of patients with New Year's Eve-associated ORL injuries in the catchment area of the University hospital of the authors remains unrecognized in this study, as not all patients consult the emergency department on the same day and are, therefore, not described in this analysis.

Strengths of the present study include the first evaluation of an ENT-specific emergency care setting on New Year's Eve and, in addition, an examination of the impact of restrictions under pandemic conditions.

Since 14 turns of the year were evaluated before the influence of the COVID-19 pandemic and only two turns of the year were analyzed under the influence of the pandemic, a statistical comparison is not sufficiently possible. Nevertheless, the descriptive analysis shows that, among other things, due to the ban on the transfer of fireworks at the turn of the years 2020/2021 and 2021/2022, no emergency presentation was required due to an ENT injury caused by fireworks. Both the average number of New Year's Eve-associated emergency patients per year and the average total number of patients during the period studied were reduced by more than half compared with previous years. The results of a retrospective analysis from 2004 about serious eye and adnexal injuries from fireworks in Northern Ireland before and after the lifting of the firework ban confirmed, also, that the removal of the legislative ban on fireworks in 1996 has had a significant effect on the incidence of eye injuries [3]. All these results should support the responsible use of fireworks even after the special restrictions of the COVID-19 pandemic have ended. This applies not only to the persons actively lighting the fireworks, but especially to bystanders and primarily minors. Evaluations from ophthalmology show that up to 60% of those injured were bystanders rather than the active igniters of the fireworks [6, 14]. Sandvall et al. found that firework shells and mortars disproportionately cause permanent impairment from eye and hand injury [17]. Concerning firework of category F2 in general, a noise level of 120 dB at a distance of 8 m may not only indicate the aforementioned acute injuries, but also it can be assumed that in some of the patients evaluated here, long-term consequences remained, which include inner ear damage with reduction of the hearing threshold and tinnitus or permanent perforation of the eardrum.

## Conclusion

The results show that New Year's Eve-associated ear, nose, and throat injuries have a wide spectrum from inner ear trauma to midface fractures. Long-term damage may include hearing loss and tinnitus. This study shall support the responsible use of fireworks even after the end of the special regulations of the COVID-19 pandemic.

## Data Availability

The authors confirm that the data supporting the findings of this study are available within the article. In addition to that, missing data are available from the corresponding author, upon request.

## Declarations

### Study approval statement

This study protocol was reviewed and approved by the local ethics committee (Ethikkommission der Universität Ulm), approval number: 176/22.
